# Microvascular obstruction is associated with greater infarct heterogeneity

**DOI:** 10.1186/1532-429X-15-S1-O74

**Published:** 2013-01-30

**Authors:** Idan Roifman, Nilesh R Ghugre, Mohammad I Zia, Alexander W Leber, Anna Zavodni, Kim A Connelly, Graham Wright

**Affiliations:** 1 Medicine, Imaging Research Centre for Cardiac Interventions, Schulich Heart Centre, Sunnybrook Health Sciences Centre, University of Toronto, Toronto, ON, Canada; 2 Medicine, St. Michael's Hospital, University of Toronto, Toronto, ON, Canada

## Background

Microvascular obstruction (MVO) has been shown to be an independent predictor of left ventricular dysfunction and death. Infarct heterogeneity as measured by gray zone has been shown to be an independent predictor of post infarct mortality. We sought to compare the evolution of gray zone in patients with and without MVO.

## Methods

21 patients post primary percutaneous coronary intervention for acute ST elevation myocardial infarction were prospectively enrolled and each had 3 MRI scans on a 1.5T GE Signa Excite scanner at 48 hours, 3 weeks and 6 months post infarction. A T1-weighted IR-GRE sequence was used for late gadolinium enhancement assessment of infarcted myocardium. Core infarct size (CIS) and gray zone (GZ) volumes were quantified using the full width at half-maximum technique and were expressed in grams. Repeated Measures Analysis of Variance was used to assess for changes in CIS and GZ evolution over the three time frames. Data is presented as means ± standard deviation at the 48 hour, 3 week and 6 month time frames respectively.

## Results

**Figure 1:**
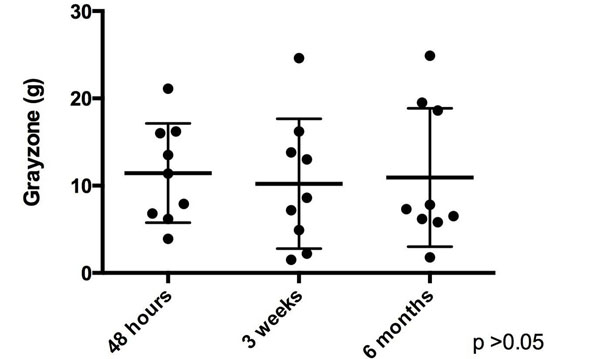
Evolution of gray zone in MVO patient s

**Figure 2:**
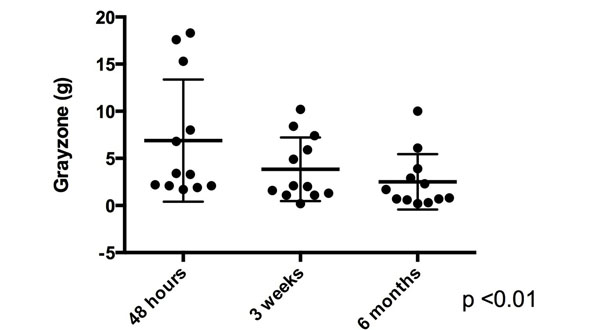
Evolution of gray zone in non-MVO patients

Of the 21 patients, 9 had evidence of MVO and 12 did not. Mean age was 57.7 ± 10.3 years. 19/21 of the patients were male. 8 of the infarcts involved the left main/left anterior descending artery, 5 involved the left circumflex artery and 8 involved the right coronary artery. Both MVO (29.6 ± 11.5 g, 15.9 ± 5.6 g, 15.2 ±7.39 g , p <0.01) and non-MVO (9.4 ± 7.7 g, 5.5 ± 4.05 g, 4.1 ± 3.5 g, p = 0.01) patients had a significant decrease in core infarct size over the three time frames. Patients with MVO did not have a significant change in gray zone across the three time points (11.5 ± 5.4 g, 10.2 ± 7.4 g and 10.9 ± 7.5 g, p = 0.83, see figure 1). In contrast, non-MVO patients did have a significant decrease of gray zone over time (6.9 ± 6.5 g, 3.8 ± 3.38 g, 2.5 ± 2.93 g, p <0.01, see figure 2).

## Conclusions

In contrast to those patients without MVO, patients with MVO did not exhibit a significant reduction in GZ. As GZ has been shown to be associated with arrhythmic events, its relative persistence over time in the presence of MVO may partially explain the mechanism leading to the poorer prognosis of MVO.

## Funding

Heart and Stroke Foundation of Canada.

Ontario Research Fund.

Canadian Institutes of Health Research.

